# A comparison of three methods in categorizing functional status to predict hospital readmission across post-acute care

**DOI:** 10.1371/journal.pone.0232017

**Published:** 2020-05-07

**Authors:** Chih-Ying Li, Amol Karmarkar, Yong-Fang Kuo, Hemalkumar B. Mehta, Trudy Mallinson, Allen Haas, Amit Kumar, Kenneth J. Ottenbacher

**Affiliations:** 1 Department of Occupational Therapy, University of Texas Medical Branch, Galveston, Texas, United States of America; 2 Division of Rehabilitation Sciences, University of Texas Medical Branch, Galveston, Texas, United States of America; 3 Department of Preventive Medicine & Community Health, University of Texas Medical Branch, Galveston, Texas, United States of America; 4 Sealy Center on Aging, University of Texas Medical Branch, Galveston, Texas, United States of America; 5 Department of Surgery, University of Texas Medical Branch, Galveston, Texas, United States of America; 6 Department of Clinical Research and Leadership, The George Washington University, Washington, DC, United States of America; 7 Department of Physical Therapy, Northern Arizona University, Phoenix, Arizona, United States of America; Texas A&M University, UNITED STATES

## Abstract

**Background:**

Methods used to categorize functional status to predict health outcomes across post-acute care settings vary significantly.

**Objectives:**

We compared three methods that categorize functional status to predict 30-day and 90-day hospital readmission across inpatient rehabilitation facilities (IRF), skilled nursing facilities (SNF) and home health agencies (HHA).

**Research design:**

Retrospective analysis of 2013–2014 Medicare claims data (N = 740,530). Data were randomly split into two subsets using a 1:1 ratio. We used half of the cohort (development subset) to develop functional status categories for three methods, and then used the rest (testing subset) to compare outcome prediction. Three methods to generate functional categories were labeled as: Method I, percentile based on proportional distribution; Method II, percentile based on change score distribution; and Method III, functional staging categories based on Rasch person strata. We used six differentiation and classification statistics to determine the optimal method of generating functional categories.

**Setting:**

IRF, SNF and HHA.

**Subjects:**

We included 130,670 (17.7%) Medicare beneficiaries with stroke, 498,576 (67.3%) with lower extremity joint replacement and 111,284 (15.0%) with hip and femur fracture.

**Measures:**

Unplanned 30-day and 90-day hospital readmission.

**Results:**

For all impairment conditions, Method III best predicted 30-day and 90-day hospital readmission. However, we observed overlapping confidence intervals among some comparisons of three methods. The bootstrapping of 30-day and 90-day hospital readmission predictive models showed the area under curve for Method III was statistically significantly higher than both Method I and Method II (all paired-comparisons, p<.001), using the testing sample.

**Conclusions:**

Overall, functional staging was the optimal method to generate functional status categories to predict 30-day and 90-day hospital readmission. To facilitate clinical and scientific use, we suggest the most appropriate method to categorize functional status should be based on the strengths and weaknesses of each method.

## Introduction

In many disciplines of medicine, clinical staging refers to hierarchical categories along the continuum of the measured construct. [1–3] The concept of “clinical staging” is also applied in acute and post-acute prospective payment systems, for example, the skilled nursing facilities (SNFs) resource utilization groups, known as case-mix group [4,5]. Individuals in the same SNFs resource utilization group are expected to share common abilities, respond similarly to assessment items, and likely have analogous needs for resources or equivalent costs of care [4,5]. When applied to functional status, known as “functional staging”, such categorizations allow clinicians to accurately plan care, track prognosis, and enable researchers to define and refine case-mix adjustment groups. Functional staging can also be used to examine intervention effectiveness [6–10], enables meaningful categorical comparisons within and across groups of person(s) and setting(s).

While continuous scores may provide detailed clinically information for clinicians [11,12], categorizing scores facilitates policy discussion and decision-making. Additionally, using continuous score produces a summed score. The same summed score could, in fact, represents different levels of performances [13]. The site-neutral unified payment model, proposed by the Medicare Payment Advisory Commission [14], recommends eliminating payment difference across settings for patients with similar case-mix demographics and severity of impairments. Generating categories based on functional status provides clinical evidence for unified payment models and other health reform measures. Investigators have demonstrated that adding functional status categories in risk-adjustment models (e.g., hospital readmission) reduces differences in population-level case-mix [15,16]. Adding functional status categories in predictive models can therefore improve the equality of resources allocation, care quality, and generate more accurate estimated care costs [17].

Practitioners and researchers have used functional status categories to present hierarchical levels of patients’ function for decades [6–10]. However, methods used to categorize functional status to predict health outcomes often are arbitrary and vary significantly. To identify the optimal method to categorize functional status, we compared three approaches in developing functional status categories to predict hospital readmission. Method I is a conventional percentile approach: tertile, quartile or quintile based on summed-scores distribution. Method II is a combination of change score with percentile method: tertile, quartile or quintile of change score between admission and discharge. Method III is a functional staging method using person strata categories based on latent trait theory. This paper aims to examine the relatively optimal approach to categorize functional status with outcome prediction in hospital readmission for Medicare beneficiaries. Hospital readmission was chosen as the main outcome in this study because it is an important national quality measure of patient care [4,18].

## Materials and methods

### Data source

The study included 100% Medicare claims data from 2013–2014. We used the following data files: Inpatient Rehabilitation Facility (IRF) and Inpatient Rehab Facility- Patient Assessment Instrument (IRF-PAI) [19]; Skilled Nursing Facility (SNF) and Minimum Data Set (MDS 3.0) [20]; Home Health Agency (HHA) and Outcome and Assessment Information Set (OASIS-C) [21]; the Medicare Provider Analysis and Review and the Master Beneficiary Summary files.

### Ethical assurances

This study was approved by the University Institutional Review Board (IRB # 16–0014). Additionally, a Data Use Agreement was established with the Centers for Medicare and Medicaid Services prior to all data analyses.

### Cohort selection

We identified 2,953,006 eligible cases using a combination of Medical Severity Diagnosis Related Group codes and ICD-9 procedure codes for three impairment conditions: stroke (061–066), lower extremity joint replacement (469–470, 81.51 and 81.54) and hip/femur fractures (480–482). Using a combination of claims and assessment data, we included only those beneficiaries discharged from a hospital to one of the three post-acute care (PAC) settings: IRF, SNF and HHA. After applying exclusion criteria (S1 Table), the final analytical sample included 740,530 cases: 17.7% with stroke (n = 130,670), 67.3% with lower extremity joint replacement (n = 498,576), and 15.0% (n = 111,284) with hip and femur fracture (Table 1).

**Table 1:** Demographics and person-level characteristics (N = 740,530).

*Discharge Locations*	All (N = 740,530)			Stroke (n = 130,670)			Lower Extremity Joint Replacement (n = 498,576)			Hip and Femur Fracture (n = 111,284)		
	IRF	SNF	HHA	IRF	SNF	HHA	IRF	SNF	HHA	IRF	SNF	HHA
***Sample Size***	137,527	325,708	277,295	56,553	41,032	33,085	52,823	210,973	234,780	28,151	73,703	9,430
Age, Mean (SD)	79.0(7.5)	79.5(7.7)	74.3(6.2)	78.8(7.5)	82.4(7.6)	80.4(7.7)	78.3(7.3)	77.5(7.0)	73.4(5.4)	80.1(7.6)	83.3(7.6)	77.3(7.8)
**Gender**												
Male	49,626 (36.1)	89,454(27.5)	114,307 (41.2)	25,974 (45.9)	15,228 (37.1)	13,237 (40.0)	16,056 (30.4)	57,327 (27.2)	98,258 (41.9)	7,596 (27.0)	16,899 (22.9)	2,812 (29.8)
Female	87,901 (63.9)	236,254 (72.5)	162,988 (58.8)	30, 579 (54.1)	25,804 (62.9)	19,848 (60.0)	36,767 (69.6)	153,646 (72.8)	136,522 (58.2)	20,555 (73.0)	56,804 (77.1)	6,618 (70.2)
**Race**												
Non-Hispanic White	116,390 (84.6)	288,288 (88.5)	247,947 (89.4)	45,267 (80.0)	33,509 (81.7)	26,386 (79.8)	46,103 (87.3)	187,580 (88.9)	231,149 (90.8)	25,020 (88.9)	67,199 (91.2)	8,412 (89.2)
Black	10,241 (7.5)	18,288 (5.6)	13,679 (4.9)	6,466 (11.4)	4,414 (10.8)	3,717 (11.2)	2,919 (5.5)	11,769 (5.6)	9,656 (4.1)	856 (3.0)	2,105 (2.9)	306 (3.2)
Hispanic	6,747 (4.9)	10,724 (3.3)	8,909 (3.2)	2,781 (4.9)	1,693 (4.1)	1,759 (5.3)	2,507 (4.8)	6,580 (3.12)	6,726 (2.9)	1,459 (5.2)	2,451 (3.3)	424 (4.5)
Others	4,149(3.0)	8,408(2.6)	6,760(2.4)	2,039(3.6)	1,416(3.5)	1223(3.7)	1,294(2.5)	5,044(2.4)	5,249(2.2)	816(2.9)	1,948(2.6)	288(3.1)
**Total IRF Stay within 90 Days**	11.8(5.6)	0.0(0.3)	0.0(0.2)	12.9(6.8)	0.0(0.5)	0.0(0.4)	10.3(4.0)	0.0(0.2)	0.0(0.1)	12.4(4.7)	0.0(0.3)	0.0(0.3)
**Total SNF Stay within 90 Days**	0.3(3.4)	22.1(17.5)	0.0(0.8)	0.4(4.0)	25.0(20.6)	0.1(1.6)	0.1(2.3)	18.2(13.2)	0.0(0.5)	0.3(3.6)	31.7(22.0)	0.1(1.7)
**Total HH Stay within 90 Days**	19.8(23.9)	14.4(18.7)	22.9(13.4)	17.3(24.1)	13.5(20.6)	28.6(18.9)	19.3(22.0)	14.0(17.7)	21.8(11.9)	25.7(25.8)	16.1(20.2)	29.8(17.9)
**Days without staying in IRF, SNF, HHA or long-term care**	37.5(31.8)	36.1(30.6)	60.3(21.8)	32.8(32.5)	18.1(25.9)	41.6(27.6)	46.4(30.4)	46.4(28.8)	63.5(19.0)	30.0(28.9)	16.8(22.8)	45.8(25.7)
**Stay in a Hospital/SNF (days)**	4.2(2.5)	4.4(2.6)	2.9(1.5)	4.4(2.8)	5.7(4.1)	3.3(2.3)	3.8(2.1)	3.8(1.9)	2.7(1.2)	4.7(2.3)	5.2(2.7)	4.3(2.6)
**Intensive Care (days)**	0.9(2.1)	0.5(1.7)	0.2(0.9)	1.7(2.5)	1.8(3.1)	1.0(1.8)	0.3(1.4)	0.2(1.0)	0.1(0.5)	0.5(1.7)	0.5(1.8)	0.3(1.3)
**Coronary Care (days)**	0.3(1.2)	0.2(1.0)	0.1(0.5)	0.5(1.5)	0.6(1.8)	0.4(1.2)	0.1(0.8)	0.1(0.6)	0.0(0.3)	0.2(1.1)	0.2(1.1)	0.1(0.7)
**Hierarchical Condition Category Score**	1.2(0.7)	1.0(0.7)	0.7(0.5)	1.4(0.7)	1.6(0.8)	1.3(0.7)	1.0(0.6)	0.9(0.6)	0.6(0.4)	1.2(0.7)	1.3(0.8)	1.0(0.8)
**Comorbidity (based on Elixhauser Comorbidity Index)**	3.4(1.9)	3.0(1.9)	2.2(1.6)	3.9(1.8)	4.2(1.9)	3.6(1.8)	2.9(1.7)	2.6(1.7)	2.0(1.4)	3.1(1.8)	3.3(1.9)	2.5(1.7)
0	4,513 (3.3)	17,826 (5.5)	30,294 (10.9)	423 (0.8)	288 (0.7)	381 (1.2)	2,833 (5.4)	14,671 (7.0)	28,950 (12.3)	1,257 (4.5)	2,867 (3.9)	963 (10.2)
1–3	75,148 (54.6)	197,692 (60.7)	196,382 (70.8)	24,839 (43.9)	16,286 (39.7)	17,156 (51.9)	33,597 (63.6)	140,339 (66.5)	173,030 (73.7)	16,712 (59.4)	41,067 (55.7)	6,196 (65.7)
4–6	49,333 (35.9)	95,028 (29.2)	46,575 (16.8)	26,007 (46.0)	19,553 (47.7)	13,293 (40.2)	14,529 (27.5)	50,357 (23.9)	31,257 (13.3)	8,797 (31.3)	25,118 (34.1)	2025 (21.5)
≥7	8,533 (6.2)	15,162 (4.7)	4,044 (1.5)	5,284 (9.3)	4,905 (12.0)	2,255 (6.8)	1,864 (3.5)	5,606 (2.7)	1,543 (0.7)	1,385 (4.9)	4,651 (6.3)	246 (2.6)
** Diagnosis**												
*Stroke*												
Ischemic	50,549 (36.8)	36,168 (11.1)	30,168 (10.9)	50,549 (89.4)	36,168 (88.2)	30,168 (91.2)						
Hemorrhagic	6,004 (4.4)	4,864 (1.5)	2,917 (1.1)	6,004 (10.6)	4,864 (11.9)	2,917 (8.8)						
*Lower Extremity Joint Replacement*												
Elective	11,273 (8.2)	52,301 (16.1)	73,190 (26.4)				11,273 (21.3)	52,301 (24.8)	73,190 (31.1)			
Non-Elective	19,333 (14.1)	44,036 (13.5)	6,443 (2.3)				19,333 (36.6)	44,036 (20.1)	6,443 (2.7)			
Knee	21,779 (15.8)	113,345(34.8)	154,205 (55.6)				21,779 (41.2)	113,345 (53.7)	154,205 (65.7)			
Others	438(0.3)	1,291(0.4)	942(0.3)				438(0.8)	1,291(0.6)	942(0.4)			
*Hip and Femur Fracture*												
Femur	2,522(1.8)	7320(2.3)	948(0.3)							2,522(9.0)	7,320(9.9)	948(10.1)
Femur Neck	24,886 (18.1)	64,112 (19.7)	7,672 (2.8)							24,886 (88.4)	64,112 (87.0)	7,672 (81.4)
Complications	463(0.3)	1,543(0.5)	492(0.2)							463(1.6)	1,543(2.1)	492(5.2)
Others	280(0.2)	728(0.2)	318(0.1)							280(1.0)	728(1.0)	318(3.4)
**Disability at Original Entitlement**												
Yes	13,608 (9.9)	28,709 (8.8)	22,231 (8.0)	5,895 (10.4)	4,052 (9.9)	3,629 (11.0)	5,216 (9.9)	18,685 (8.9)	17,654 (7.5)	2,497 (8.9)	5,942 (8.1)	948 (10.1)
No	123,919 (90.1)	296,999 (91.2)	255,064 (92.0)	50,658 (89.6)	36,980 (90.1)	29,456 (88.0)	47,607 (90.1)	192,288 (91.1)	217,126 (92.5)	25,654 (91.1)	67,731 (91.9)	8,482 (90.0)
**Medicaid Eligibility**												
Yes	17,952 (13.1)	43,417 (13.3)	18,385 (6.6)	9,025 (15.9)	9,222 (22.5)	5,732 (17.3)	5,345 (10.1)	22,053 (10.5)	11,443 (4.9)	3,582 (12.7)	12,142 (16.5)	1,210 (12.8)
No	119,575 (86.9)	282,291 (86.7)	258,910 (93.4)	47,528 (84.0)	31,810 (77.5)	27,353 (82.7)	47,478 (89.9)	188,920 (89.6)	223,337 (95.1)	24,569 (87.3)	61,561 (83.5)	8,220 (87.2)
**30-Day Readmission**	14,995 (10.9)	30,815 (9.5)	9,803 (3.5)	7,833 (13.9)	6,931 (16.9)	2,413 (7.3)	4,338 (8.2)	14,605 (6.9)	6,984 (3.0)	2,824 (10.0)	9,279 (12.6)	406 (4.3)
**90-Day Readmission**	26,210 (19.1)	58,407 (17.9)	22,298 (8.0)	13,723 (24.7)	13,222 (33.2)	5,556 (16.8)	7,635 (14.5)	27,526 (13.1)	15,758 (6.7)	4,852 (17.2)	17,659 (24.0)	984 (10.4)

IRF = inpatient rehabilitation facility; SNF = skilled nursing facility; HHA = home health agency.

To develop and validate the three proposed methods, we used 1:1 ratio to randomly split the study cohort into a development subset (n = 370,265) and a testing subset (n = 370,265). The development subset was used to develop functional status categories from three methods. The testing subset was used to compare outcome prediction for three methods.

We also conducted sensitivity analysis to examine difference of demographics and person-level characteristics before and after excluding 23% of potential patients (step 12 vs. step 15 in S1 Table). The cohort in step 12 included patients who did not receive PAC. The cohort that included 23% patients (generated by step 12) had less total SNF stay within 90 days at IRF compared to the cohort used in this study (generated by step 15). However, we did not find other variables significantly different between step-12 cohort and our study cohort (S6 Table).

### Study outcome

The primary outcome was unplanned all-cause 30-day and 90-day hospital readmission (yes/no) after index hospital discharge [22,18]. We chose 30-day window to reflect current reimbursement system. Additionally, we included a longer follow-up time-period (90-day) to be consistent with the episode-based payment initiatives [23,24].

### Primary variable

The primary variable was functional status categories for two domains (Self-Care and Mobility) generated from three methods (details below). Self-Care and Mobility domains were chosen as these two domains being consistently measured across the PAC settings. Additionally, these two domains are potentially modifiable factors relevant to hospital readmission.

### Functional status categories

Comparable items of the Self-Care and Mobility domains from each assessment were selected based on their conceptual meanings (e.g., eating items were selected from IRF-PAI, MDS and OASIS as the three items measure the same activity: eating). The number of selected items by assessment was 11 in IRF-PAI (6 Self-Care and 5 Mobility items), 11 in MDS (5 Self-Care and 6 Mobility) and 8 in OASIS (5 Self-Care and 3 Mobility) (S2 Table). We used co-calibration tables [25] to co-calibrate Self-Care and Mobility scores separately into a 0–100 scale, for the following three methods.

#### Method I: Percentile based on proportional distribution

For each impairment condition, we created tertile, quartile and quintile categories based on the co-calibrated summed score distribution for each assessment. Self-Care and Mobility had the same numbers of categories. We generated percentiles first for each assessment, following c-statistics to determine whether to choose tertile, quartile or quintile for each impairment condition at each setting. Based on the c-statistics, quartile was chosen for stroke and lower extremity joint replacement, and quintile was chosen for hip and femur fracture. S1 Fig demonstrates an example of using Method I to generate functional categories of IRF-PAI Self-Care in Stroke. The same procedure was repeated for MDS and OASIS across impairment conditions. Detailed categories were provided in S3 Table.

#### Method II: Change score with percentile distribution

We first calculated the change score between admission and discharge for each assessment (Self-Care and Mobility were calculated separately). Secondly, we calculated percentile (tertile, quartile and quintile) based on the change score distribution. Lastly, to increase clinical meaningfulness when interpreting negative, zero and positive change scores, we combined the percentile change score distribution with the following operational definitions: tertiles (small, medium and large change), quartiles (negative and zero change, small positive change, medium positive change and large positive change) and quintiles (negative change, zero change, small positive change, medium positive change and large positive change).

Same as Method I, Self-Care and Mobility of each assessment had the same number of categories due to the nature of percentile method. Using c-statistics, quartile was selected for stroke and lower extremity joint replacement; quintile was selected for hip and femur fracture. The quintile proportion was found inapplicable for stroke and lower extremity joint replacement as the same functional score was used in more than one category. S2 Fig demonstrates an example of using Method II to generate functional categories of IRF-PAI Self-Care in Stroke. The same procedure was repeated for MDS and OASIS across impairment conditions. Detailed categories were provided in S4 Table.

#### Method III: Functional staging

**Fig 1:**
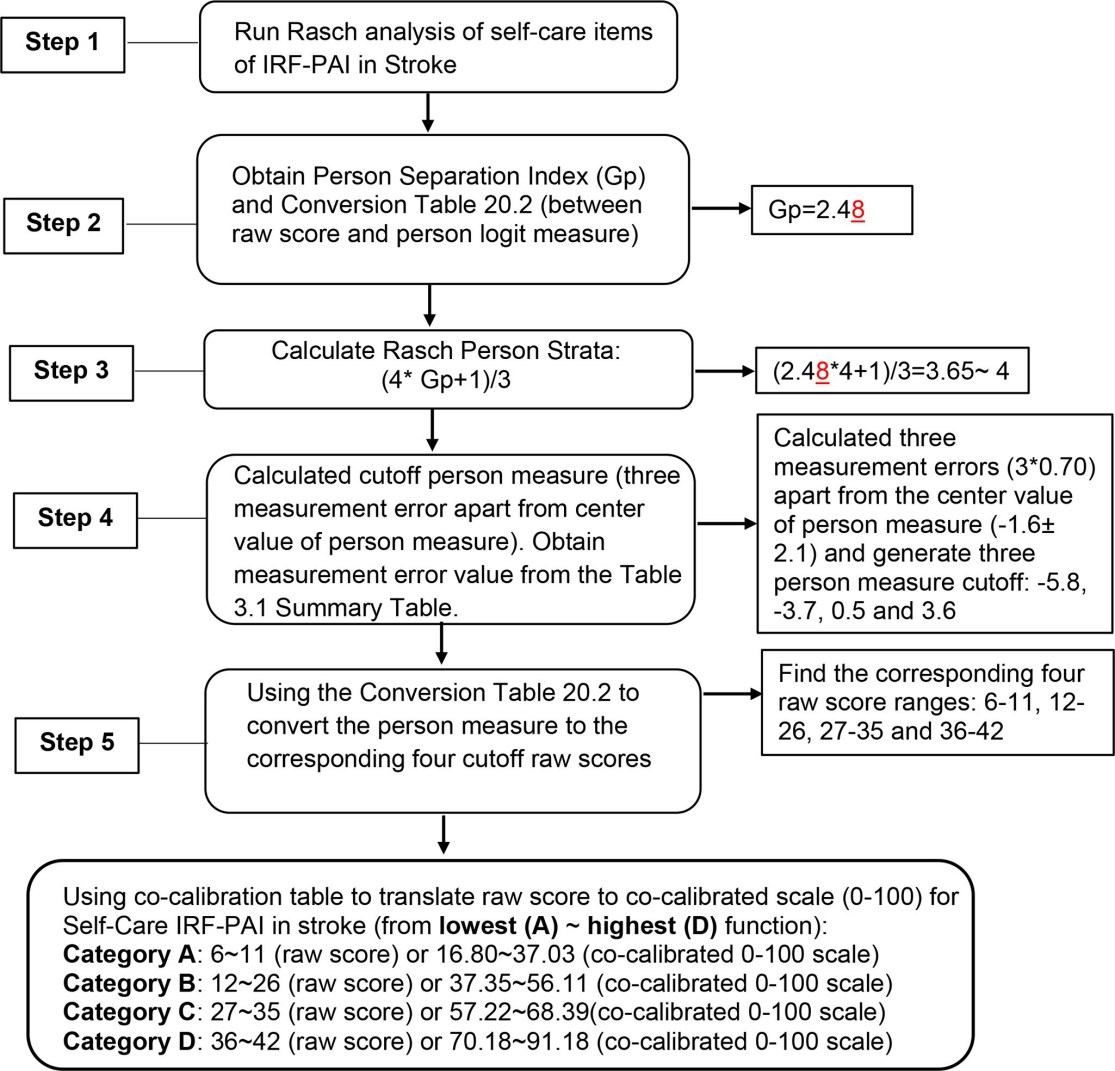
Method III: Use functional staging approach to generate functional score categories (Example of IRF-PAI Self-Care in Stroke).

Fig 1 provides the detailed procedures demonstrating how we generated functional staging categories for IRF-PAI Self-Care in Stroke. We generated a person separation index (Gp) and calculated person strata, to statistically distinguish different ability levels using Rasch person strata formula (4*Gp+1)/3 [26–31]. We followed this existing formula to calculate the number of person strata for each assessment by impairment condition [26–36]. Person strata are the concept based on a norm reference method using the distribution of person measure and centering on the mean of the person distribution. Each strata needs to be separated by at least three measurement errors apart to be statistically distinct [26–31]. We then identified the corresponding cutoff raw score from the 0–100 scale co-calibration table [25].

Using the development subset, for stroke, we generated four categories for Self-Care and three categories for Mobility for all three instruments. For lower extremity joint replacement, we generated three Self-Care and two Mobility categories for IRF-PAI and OASIS; and three Self-Care and three Mobility categories for MDS. For hip and femur fracture, we generated three Self-Care and two Mobility categories for IRF-PAI; two Self-Care and three Mobility categories for MDS; and four Self-Care and three Mobility categories for OASIS (S5 Table).

### Model comparisons

Six indices were used to compare the outcome prediction of the three methods:

#### C-statistics/Area under the Curve (AUC)

The c-statistics measure the discrimination ability of the model. We compared the logistic model discrimination using c-statistics with asymptotic 95% confidence intervals. The c-statistic is also known as the AUC, the area under the receiver operating characteristic curves. The AUC is the most commonly used method to evaluate probability of model performance in the context of binary outcomes with higher values indicates better model fit [37–42].

#### Somer’s Delta (Somer’s D)

Somer’s D is a nonparametric test to assess the strength and direction of the association between an ordinal dependent variable and an ordinal independent variable. Somer’s D is based on the assumption of a monotonic relationship between the independent and the outcome variables. Higher Somer’s D indicates better model fit [43].

#### Akaike information criterion (AIC)/Bayesian information criterion (BIC)

Both AIC and BIC [44] evaluate goodness-of-fit (model fit) and penalize for the excessive number of estimated parameters using log-likelihood functions. AIC/BIC provide a standard to balance between model parsimony and the penalty for overfitting [45,46]. Lower AIC/BIC value indicates better model fit [44,45].

#### Integrated Discrimination Improvement (IDI)

The IDI indicates the difference in discrimination slopes between two models. The IDI measures whether the new model improves the average sensitivity without sacrificing its average specificity [47]. Higher (positive) values of IDI indicate that the new model is better than the reference model.

#### Net Reclassification Improvement (NRI)

The NRI is a reclassification measure using reclassification tables constructed separately for respondents with and without events (i.e., outcome occurs or not) between two models [48]. Higher (positive) values of NRI (percent) indicate reclassification by the new model had higher sensitivity compared to the reference model.

### Statistical analyses

We stratified all analyses by impairment conditions for both development and testing subsets. First, we constructed a baseline logistic regression model which included sociodemographic variables (age, sex, race/ethnicity, disability entitlement and Medicare-Medicaid dual eligibility), health status (Hierarchical Condition Category composite score, Elixhauser comorbidity categories, condition-specific severity, hospital length of stay, intensive care days and coronary care days) and post-acute length of stay. Then, we added three types of functional status to the baseline logistic regression model. We used baseline model to (a) ensure fair comparison conveyed by different functional status categories from three methods, and to (b) examine the magnitude change of outcome prediction by adding functional status variables. The predictive models with three methods of generating functional status categories were examined by AUC, Somer’s D, AIC, BIC, IDI and NRI using the testing sample. To validate the stability of the estimates, a bootstrap procedure with 1000 re-samples was used to statistically compare c-statistics of the three methods using the testing sample. The c-statistics with bootstrapping is a standardized way for model comparison. Each of the three methodologies were later compared using paired t-tests if significant difference existed among methods. We used SAS version 9.4 (SAS Institute, Inc., Cary, NC) to perform all analyses.

## Results

### Demographics

The majority were discharged to SNF (n = 325,708; 44.0%), followed by HHA (n = 277,295; 37.4%) and IRF (n = 137,527; 18.6%) (Table 1). The mean ages were 79.0 (7.6), 79.5 (7.7) and 74.3 (6.2) at IRF, SNF and HHA, respectively. The majority were female (63.9%, 72.5% and 58.8% at IRF, SNF and HHA, respectively) and non-Hispanic White (84.6%, 88.5% and 89.4% at IRF, SNF and HHA, respectively). The most common impairment conditions across PAC settings were ischemic stroke (n = 50,549; 36.8%) at IRF; knee replacement for both SNF (n = 113,345; 34.8%) and HHA (n = 154,205; 55.6%). Patients at IRF had slightly more comorbidities [3.4 (1.9)], compared to SNF 3.0 (1.9) and HHA 2.2 (1.6). Patients discharged to IRF had the highest rate of 30-day (10.9%) and 90-day hospital readmission (19.1%), compared to SNF (9.5%, 17.9%) and HHA (3.5%, 8.0%) (Table 1).

### Model comparisons of three methods

Table 2 reports performance metric of different methods in predicting 30-days and 90-day readmissions. For all three impairment conditions, c-statistics and Somer’s D were the highest and AIC/BIC were the lowest for Method III in predicting 30-day and 90-day readmissions. For example, among patients with stroke, Method III had the highest c statistics (0.8340) compared to Methods I (0.8319) and II (0.8271) and the lowest AIC (Method III: 38442, Method II: 28958, Method I: 28615) and BIC (Method III: 38642, Method II: 39167, Method I: 38824) for 30-day hospital readmission. Three impairment conditions had the same result: Method III better predicted both 30-day and 90-day hospital readmission compared to Methods I and II (Table 2). Method III also had positive IDI and NRI values (better slope discrimination) compared to Method I and Method II (Table 2). However, the confidence intervals of Method III (both 30- and 90-day) were overlapping with those of Method I and Method II for stroke, lower extremity joint replacement and hip/femur fracture.

**Table 2:** Comparisons of outcome predictions with three functional category methods (N = 370,265).

**Outcome (1): 30-Day Readmission**				
**Stroke**				
	***Baseline***	***Method I***	***Method II***	***Method III***
**AUC**	0.8267	0.8319	0.8271	0.8340
**95% CI for AUC**	0.8229–0.8305	0.8282–0.8357	0.8233–0.8309	0.8303–0.8378
**Somer’s D**	0.6534	0.6639	0.653	0.6681
**AIC**	38978	38615	38958	38442
**BIC**	39132	38824	39167	38642
**IDI**		**Ref.**	-0.00756	0.06569
			**Ref.**	0.05826
**NRI**		**Ref.**	-0.36571	0.15765
			**Ref.**	0.10141
**Lower Extremity Joint Replacement**				
	***Baseline***	***Method I***	***Method II***	***Method III***
**AUC**	0.8684	0.8691	0.8684	0.8695
**95% CI for AUC**	0.8656–0.8712	0.8666–0.8722	0.8656–0.871	0.8667–0.8723
**Somer’s D**	0.7368	0.7390	0.7368	0.7391
**AIC**	75475	75264	75483	75176
**BIC**	75673	75504	75744	75436
**IDI**		**Ref.**	-0.0036115	0.001221
			**Ref.**	0.037935
**NRI**		**Ref.**	-0.17786	0.098408
			**Ref.**	0.15809
**Hip and Femur Fracture**				
	***Baseline***	***Method I***	***Method II***	***Method III***
**AUC**	0.8782	0.8789	0.8783	0.8792
**95% CI for AUC**	0.8751–0.8813	0.8758–0.8820	0.8752–0.8814	0.8761–0.8823
**Somer’s D**	0.7564	0.7577	0.7565	0.7585
**AIC**	26719	26687	26725	26653
**BIC**	26889	26910	26949	26867
**IDI**		**Ref.**	-0.082655	0.08079
			**Ref.**	0.07865
**NRI**		**Ref.**	-0.32637	0.25179
			**Ref.**	0.19182
**Outcome (2): 90-Day Readmission**				
**Stroke**				
	***Baseline***	***Method I***	***Method II***	***Method III***
**AUC**	0.6794	0.6842	0.6800	0.6886
**95% CI for AUC**	0.6748–0.6840	0.6796–0.6888	0.6754–0.6846	0.6840–0.6932
**Somer’s D**	0.3587	0.3683	0.3600	0.3772
**AIC**	68533	68287	68510	68041
**BIC**	68688	68496	68719	68241
**IDI**		**Ref.**	-0.03035	0.02071
			**Ref.**	0.02330
**NRI**		**Ref.**	-0.20084	0.00187
			**Ref.**	0.03031
**Lower Extremity Joint Replacement**				
	***Baseline***	***Method I***	***Method II***	***Method III***
**AUC**	0.7153	0.7171	0.7153	0.7173
**95% CI for AUC**	0.7118–0.7189	0.7135–0.7206	0.7118–0.7189	0.7137–0.7208
**Somer’s D**	0.4307	0.4341	0.4307	0.4346
**AIC**	149292	148990	149289	148982
**BIC**	149490	149251	149549	149222
**IDI**		**Ref.**	-0.00157	0.00162
			**Ref.**	0.01566
**NRI**		**Ref.**	-0.11736	0.04468
			**Ref.**	0.13081
**Hip and Femur Fracture**				
	***Baseline***	***Method I***	***Method II***	***Method III***
**AUC**	0.7426	0.7436	0.7427	0.7444
**95% CI for AUC**	0.7380–0.7473	0.7390–0.748	0.7380–0.7473	0.7397–0.7490
**Somer’s D**	0.4853	0.4872	0.4854	0.4887
**AIC**	50675	50634	50682	50582
**BIC**	50844	50857	50908	50797
**IDI**		**Ref.**	-0.04247	0.03827
			**Ref.**	0.04000
**NRI**		**Ref.**	-0.13081	0.08185
			**Ref.**	0.17813

***Method I***: Percentile summed-score distribution (quartile for stroke and lower extremity joint replacement; quintile for hip and femur fracture). ***Method II***: Percentile change score (discharge minus admission scores). ***Method III***: Functional staging method.^1^

***Abbreviations***: AUC: Area under the Curve; AIC: Akaike information criteria; BIC: Bayesian information criterion; IDI: Integrated Discrimination Index; NRI: Net Reclassification Improvement; IDI: Integrated Discrimination Index; NRI: Net Reclassification Improvement.

^1^ Table 2 presents results under the context of baseline model plus the three Methods. We constructed the baseline model with the following variables: (a) sociodemographic (age, sex, race/ethnicity, disability as the original reason for entitlement {yes/no}, Medicare-Medicaid dual eligibility {yes/no}), health status (Hierarchical Condition Category composite score, Elixhauser comorbidity categories (0, 1–3, 4–6 and ≥7 comorbidities, condition-specific severity {for stroke: hemorrhage/ischemic; for Lower Extremity Joint Replacement: non-elective hip, elective hip, knee joint replacement and others; for Hip and Femur Fracture: femur fracture, femur neck fracture, complication fracture and other}, hospital length of stay {LOS; continuous}, intensive care days used by beneficiary for stay {continuous}, and coronary care days used by beneficiary for stay {continuous}, post-acute LOS in all PAC settings {IRF, SNF and HHA; continuous}. We added functional status categories (Self-Care and Mobility) generated from each method (Methods I-III) to the baseline model.

### Bootstrapping

In both 30-day and 90-day hospital readmission models, the results of the bootstrapping using testing sample showed that the AUC for Method III was the highest compared with both Method I and Method II for the three impairment conditions (all paired-comparisons, p<.001).

### Clinical application

**Fig 2:**
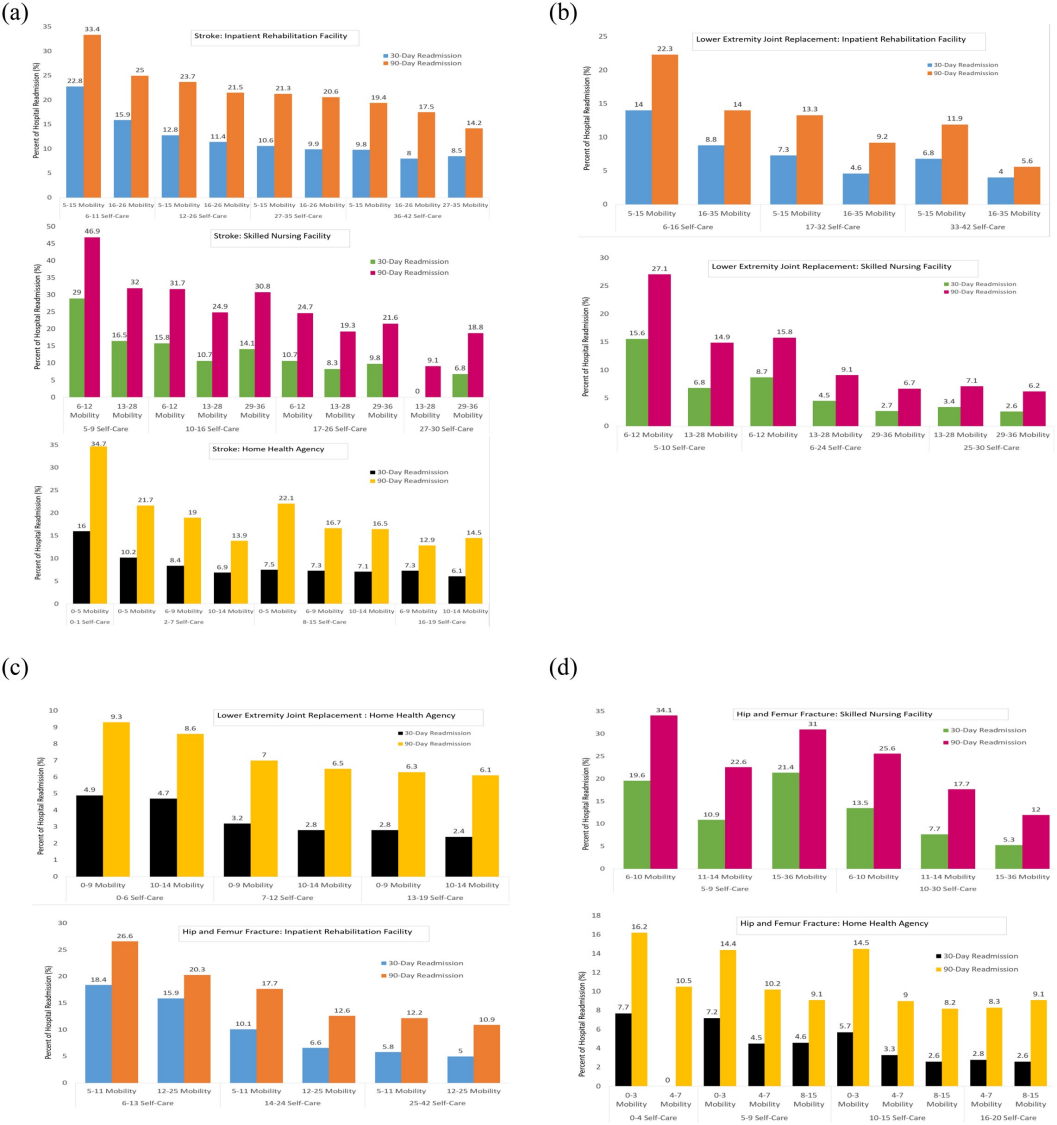
Using Method III to estimated 30-day and 90-day hospital readmission rate.

We provided functional status categories generated from Methods I-III (S3–S5 Tables). We also provide the estimated risk of 30-day and 90-day hospital readmission using the self-care and mobility combinations based on Method III functional staging categories (Fig 2). For example, among patients with stroke who had self-care score between 6–11, those with mobility score between 5–15 will have 22.8% probability of 30-day readmission and 33.4% probability of 90-day readmission (Fig 2).

## Discussion

Generating meaningful categories allow for functional status comparisons and optimal outcome prediction across post-acute settings. This study compared three functional category methods and found the functional staging approach (Method III) generated the relatively optimal prediction for 30-day and 90-day hospital readmission. While the study findings imply that using functional staging approach can be relatively optimal for outcome prediction, it is unclear whether this improvement can also produce superior clinically meaningful levels. To facilitate clinical and scientific use, we suggest the most appropriate method to categorize functional status should be based on the strengths and weaknesses of each approach. For example, Method I may have the advantage of convenience (quick to calculate), Method II may have the advantage when reporting functional change and Method III may have the advantage in outcome prediction (i.e. hospital readmission). The choice of the method requires a delicate judgement and balance between available resource, time demand and study purpose. This study provides preliminary data to guide future healthcare policy reforms (e.g., bundled payment) when classifying patients’ self-care and mobility function. We also generated tables of functional categories based on the three methods and plots of function-based readmission risks using functional staging for clinicians and researchers to use.

Policymakers are beginning to explore the impact of functional status on classification systems in post-acute risk-adjusted capitation payments [15,49,50]. Researchers and the Medicare Payment Advisory Commission reported that adding functional status improved prediction of resource use and cost of care [15–16,49–51]. Categorizing patients into clusters would be clinically and administratively useful (e.g. patients in the same cluster may experience comparable care cost or require similar resources). By its nature, functional staging is hierarchical and thus may provide gradients of functional recovery (or loss) that can help case-mix adjustment in services use and outcome comparisons, aiding in care provision, resource allocation decisions and eventually quality of care evaluation.

We acknowledge that patients with varying clinical characteristics and disease severity may benefit differently from various levels of care provided at different types of post-acute settings. However, recent healthcare reform proposals emphasize the need for a unified prospective payment system for post-acute settings [14]. Thus, comparisons of effectiveness and efficiency of care for patients with similar case-mix demographics across post-acute settings are eminent and inevitable. Identifying standardized and consistent approaches to measure functional status across post-acute settings could inform future policy decisions and improve quality of patient care after hospitalization. Based on the Improving Medicare Post-Acute Care Transformation Act of 2014, Centers for Medicare and Medicaid Services Section GG data elements were implemented to collect unified functional data across PAC settings [52,53]. While Section GG data elements potentially would resolve functional assessment issues related to uniformity across PAC settings, using a standardized functional categorization method based on co-calibration functional scores provides firsthand comparisons of functional status across PAC settings. This study serves as a basis for Section GG data elements to develop hierarchical functional categorizations across settings in the future.

The study findings also indicate that generating more categories is not associated with better outcome prediction. Our results support the notion that the number of functional status categories varies by impairment condition, and using distinct functional levels may be more appropriate than the arbitrary percentile cutoff criteria, where a predefined fixed number of distribution-based categories dictates the categorization. Functional staging consider hierarchical functional levels, thus this empirical approach can classify patients into distinct functional levels.

Current evidence regarding the advantages and limitations of different functional category methods remains unclear and largely unexplored. In the emerging environment of value-based care and precision medicine, it is reasonable to ask: are percentile proportional distribution and change score too insensitive to provide accurate functional categories necessary to assess and predict quality outcomes? If the answer is yes, then what are the appropriate approaches? Our study and findings address this question and provide a potential solution for improving rigor in comparative effectiveness studies across post-acute settings.

Ongoing demonstration projects of uniform functional assessment, episode-based payment models, and unified payment system across post-acute settings signify the growing need to conduct rigorous post-acute health services and health policy research. This study is the first we are aware of to examine the impact of quality measures based on different categorization methods of functional status. Future study should examine whether different categorization methods of functional status are associated with different provision of care services. It is also important to explore other variables in addition to functional status to optimize outcome prediction accuracy for individual patients. In addition, future study should validate whether our finding can be applied to other quality outcomes, such as successful community discharge for Medicare beneficiaries.

### Study limitations

This study has limitations related to using Medicare files [54]. For example, our findings may not be applicable to persons < 66 years old or those enrolled in insurance plans other than Fee-For-Services. In addition, this study focused on the physical aspects of functional status while cognitive function is an essential element of functional performance. We suggested future studies of this kind include cognitive function items. We are aware of the importance of stability of functional staging for both clinical application and policy decision-making, and recognize that co-calibration methodologies may introduce conversion measurement errors. We are also aware of that using categorization may introduce discontinuity at the boundaries of cut-off scores, thus limit statistical power, precision, and obscure the ‘functionality’ of individual differences. Future study also needs to identify whether the improvement of functional staging approach has clinical meanings compared to alternative methods. We also suggest future study investigating whether different clinically meaningful change levels can and/or should be included within each category, or if items should be weighted to enhance accuracy for both clinical utility and policy decision-making.

## Conclusions

Current measures and methods examining functional status across post-acute settings vary significantly. To compare effectiveness and quality of care across post-acute settings, identifying an optimal functional category method is imperative. While our study found functional staging approach generated functional categories that explained the largest variances in both 30-day and 90-day hospital readmission prediction, we are uncertain whether functional staging approach can provide clinically meaningful improvement compared to alternative methods. We suggest clinicians, researchers and policy makers execute their best judgments to balance the strengths and weaknesses of each method when categorizing functional status. Additional research is needed to better understand the advantages and the limitations of using functional staging categories to assess and predict other important national quality measures across post-acute settings.

## Supporting information

S1 Table Cohort selection criteria. (DOCX)

S2 Table Selected functional items in IRF-PAI, MDS and OASIS (Self-Care, Mobility). (DOCX)

S3 Table Method I (Percentile Admission Score): Raw scores for IRF-PAI, MDS and OASIS (Self-Care & Mobility) in stroke, lower extremity joint replacement and hip/femur fracture (Quartile). (DOCX)

S4 Table Method II (Percentile Change Score): Raw scores for IRF-PAI, MDS and OASIS (Self-Care & Mobility) in stroke, lower extremity joint replacement and hip/femur fracture (0–100 Co-calibrated Score)^. (DOCX)

S5 Table Method III (Functional Staging based on Rasch Model): Raw scores for IRF-PAI, MDS and OASIS (Self-Care & Mobility) in stroke, lower extremity joint replacement and hip/femur fracture. (DOCX)

S6 Table Demographics and person-level characteristics of inclusion and exclusion samples. (DOCX)

S1 Fig Method I: Use percentile admission score to generate functional score categories (Example of IRF-PAI Self-Care in Stroke). (DOCX)

S2 Fig Method II: Use percentile change score to generate functional score categories (Example of IRF-PAI Self-Care in Stroke). (DOCX)

## Abbreviations

HHA: Home Health Agency
IDI: Integrated Discrimination Index
IRF-PAI: Inpatient Rehab Facility- Patient Assessment Instrument
IRF: Inpatient Rehabilitation Facility
MDS: Minimum Data Set
NRI: Net Reclassification Improvement
OASIS: Outcome and Assessment Information Set
PAC: Post Acute Care
SNF: Skilled Nursing Facility
