# Supplementary material for: A comparison of three methods in categorizing functional status to predict hospital readmission across post-acute care
Source: PLoS One. 2020 May 7;15(5):e0232017. doi: 10.1371/journal.pone.0232017 (PMC7205206; doi:10.1371/journal.pone.0232017)
Supplement: S1 Table — (DOCX) [file pone.0232017.s001.docx]

**Appendix Table 1. Cohort Selection Criteria**

| Selection Criteria | N | % of previous step |
| --- | --- | --- |
| 1. Acute Hospitalization for stroke (Medical Severity Diagnosis Related Group Codes: 61-66), joint replacement (469, 470), hip and femur procedure (480, 481, 482) discharged between 1/1/2013 and 8/31/2014 | 2,953,006 | - |
| 2. Select the 1st discharge for each patient in each DRG cluster (stroke, joint replacement, hip and femur procedure) | 2,727,313 | 92.4% |
| 3. Age 66-100 at admission | 2,375,845 | 87.1% |
| 4. Complete Part A coverage in the prior year and 90 days after discharge or till death, whichever was earlier | 2,130,625 | 89.6% |
| 5. No Health Maintenance Organization insurance enrollment plans in the prior year and 90 days after discharge or till death, whichever was earlier | 1,479,049 | 69.4% |
| 6. Not transferred from SNF/ long-term care nursing home and admission is either Elective, Urgent or Emergency | 1,391,711 | 94.1% |
| 7. Exclude those transferred to another hospital | 1,374,809 | 98.8% |
| 8. Keep only patients who had an IRF stay within 3 days, a SNF stay within 8 days, a HHA claim within 10 days or discharged to self-care. | 1,295,137 | 94.2% |
| 9. Remove patients with an IRF, SNF or HHA stay as described in step 8 but had an additional acute stay before it. | 1,291,528 | 99.7% |
| 10. Remove patients with an IRF, SNF or HHA stay immediately preceding their index acute stay. | 1,240,696 | 96.1% |
| 11. Remove patients who have long-term care stay of at least 30 days during follow-up. | 1,232,216 | 99.3% |
| 12. Remove patients who did not receive PAC care | 1,001,428 | 81.3% |
| 13. Remove patients who have missing or incomplete assessments, or whose assessment data is otherwise problematic (i.e. large gaps between last assessment and discharge) at admission and discharge. | 776,881 | 77.6% |
| 14. Remove patients who have claims overlap. | 740,846 | 95.4% |
| 15. Remove patients who have negative co-calibrated functional scores. | 740,530 | 99.9% |

HHA=home health; IRF= inpatient rehabilitation facility; SNF=skilled nursing facility; PAC=post-acute care; MDS=Minimum Data Set.
