# Supplementary material for: A comparison of three methods in categorizing functional status to predict hospital readmission across post-acute care
Source: PLoS One. 2020 May 7;15(5):e0232017. doi: 10.1371/journal.pone.0232017 (PMC7205206; doi:10.1371/journal.pone.0232017)
Supplement: S2 Table — (DOCX) [file pone.0232017.s002.docx]

**Appendix Table 2. Selected Functional Items in IRF-PAI, MDS and OASIS (Self-Care, Mobility).**

| **Domains** | **IRF-PAI (N=11)** | **MDS-3.0 (N=11)** | **OASIS (N=8)** |
| --- | --- | --- | --- |
| **Self-Care** | Item #39 (A-F) (n=6):  Eating, grooming, bathing, dressing-upper, dressing-lower, toileting | Section 110 (G1-J2) & 120AB (n=5):  Toileting, dressing eating, grooming/personal hygiene, bathing | Section K (M1800, M1810, M1820, M1830, M1845, M1870) (n=5): Grooming, dress upper body, dress lower body, bathing, feeding or eating |
| **Mobility (including transfer)** | Item #39 (I-M) (n=5):  Bed/chair/wheelchair, toilet, tub/shower, walk/wheelchair, stairs | Section 110 (A1-F2) (n=6):  Bed mobility, Transfer (bed/chair/wheelchair), walk in room, walk in corridor, locomotion on unit, locomotion off unit | Section K (M1840, M1850, M1860) (n=3):  Toilet transferring, transferring (bed/chair), ambulation/locomotion |
| **Rating Scale** | 1= Total Assistance 2= Maximal Assistance (>25% independence) 3= Moderate Assistance (>50% independence) 4= Minimal Assistance (>75% independence) 5= Supervision 6= Modified Independence 7= Complete Independence | **Original Rating Score**^#^**:**  0 = Independent  1 = Supervision  2 = Limited Assistance  3 = Extensive Assistance  4 = Total dependence | **Original Rating Score:**  0=Total independent  1=Supervision/limited assistance  2=Assistance  3=Total dependence |

IRF-PAI=Inpatient Rehabilitation Facility Patient Assessment Instrument; MDS=Minimum Data Set; OASIS=Outcome and Assessment Information Set.

^#^Self-performance and support items were used to generate a combined score (0-6) for each MDS item (original score is 0-4 as listed above). Detailed MDS score reversing process of each item could be obtained upon requested.
