# Supplementary material for: A comparison of three methods in categorizing functional status to predict hospital readmission across post-acute care
Source: PLoS One. 2020 May 7;15(5):e0232017. doi: 10.1371/journal.pone.0232017 (PMC7205206; doi:10.1371/journal.pone.0232017)
Supplement: S3 Table — (DOCX) [file pone.0232017.s003.docx]

**Appendix Table 3. Method I (Percentile Admission Score): Raw Scores for IRF-PAI, MDS and OASIS (Self-Care & Mobility) in Stroke, Lower Extremity Joint Replacement and Hip/Femur Fracture (Quartile).**

| **Stroke** | | | | | | | | | | | |
| --- | --- | --- | --- | --- | --- | --- | --- | --- | --- | --- | --- |
| **IRF-PAI** | | | | **MDS** | | | | **OASIS** | | | |
| **Self-care** | | **Mobility** | | **Self-care** | | **Mobility** | | **Self-care** | | **Mobility** | |
| **Category** | **Raw Score** | **Category** | **Raw Score** | **Category** | **Raw Score** | **Category** | **Raw Score** | **Category** | **Raw Score** | **Category** | **Raw Score** |
| A* | 6-14 | A | 5-8 | A | 5-9 | A | 6-8 | A | 0-9 | A | 0-8 |
| B | 15-20 | B | 9-12 | B | 10-12 | B | 9-11 | B | 10-11 | B | 9 |
| C | 21-25 | C | 13-15 | C | 13-15 | C | 12-16 | C | 12-13 | C | 10-11 |
| D | 26-42 | D | 16-36 | D | 16-30 | D | 17-36 | D | 14-19 | D | 12-14 |
| **Lower Extremity Joint Replacement** | | | | | | | | | | | |
| **IRF-PAI** | | | | **MDS** | | | | **OASIS** | | | |
| **Self-care** | | **Mobility** | | **Self-care** | | **Mobility** | | **Self-care** | | **Mobility** | |
| **Category** | **Raw Score** | **Category** | **Raw Score** | **Category** | **Raw Score** | **Category** | **Raw Score** | **Category** | **Raw Score** | **Category** | **Raw Score** |
| A | 6-18 | A | 5-8 | A | 5-13 | A | 6-10 | A | 0-10 | A | 0-8 |
| B | 19-22 | B | 6-11 | B | 14 | B | 11-14 | B | 11 | B | 9 |
| C | 23-25 | C | 12-13 | C | 15-17 | C | 15-18 | C | 12 | C | 10 |
| D | 26-42 | D | 14-36 | D | 18-30 | D | 19-36 | D | 13-19 | D | 11-14 |
| **Hip and Femur Fracture** | | | | | | | | | | | |
| **IRF-PAI** | | | | **MDS** | | | | **OASIS** | | | |
| **Self-care** | | **Mobility** | | **Self-care** | | **Mobility** | | **Self-care** | | **Mobility** | |
| **Category** | **Raw Score** | **Category** | **Raw Score** | **Category** | **Raw Score** | **Category** | **Raw Score** | **Category** | **Raw Score** | **Category** | **Raw Score** |
| A | 6-15 | A | 5-7 | A | 5-9 | A | 6-7 | A | 0-9 | A | 0-8 |
| B | 16-18 | B | 8-10 | B | 10-11 | B | 8-10 | B | 10 | B | 9 |
| C | 19-21 | C | 11-13 | C | 12-13 | C | 11-12 | C | 11-12 | C | 10 |
| D | 22-24 | D | 14-16 | D | 14-16 | D | 13-18 | D | 13-14 | D | 11 |
| E | 25-42 | E | 17-35 | E | 17-30 | E | 19-36 | E | 15-19 | E | 12-14 |

*: A represents the lowest functional group. D/E represents the highest functional group. IRF-PAI=Inpatient Rehabilitation Facility Patient Assessment Instrument; MDS=Minimum Data Set; OASIS=Outcome and Assessment Information Set.
