# Supplementary material for: A comparison of three methods in categorizing functional status to predict hospital readmission across post-acute care
Source: PLoS One. 2020 May 7;15(5):e0232017. doi: 10.1371/journal.pone.0232017 (PMC7205206; doi:10.1371/journal.pone.0232017)
Supplement: S4 Table — (DOCX) [file pone.0232017.s004.docx]

**Appendix Table 4. Method II (Percentile Change Score): Raw Scores for IRF-PAI, MDS and OASIS (Self-Care & Mobility) in Stroke, Lower Extremity Joint Replacement and Hip/Femur Fracture (0-100 Co-calibrated Score)^**

| **Stroke** | | | | | | | | | | | |
| --- | --- | --- | --- | --- | --- | --- | --- | --- | --- | --- | --- |
| **IRF-PAI** | | | | **MDS** | | | | **OASIS** | | | |
| **Self-care** | | **Mobility** | | **Self-care** | | **Mobility** | | **Self-care** | | **Mobility** | |
| **Category** | **Raw Change Score** | **Category** | **Raw Change Score** | **Category** | **Raw Change Score** | **Category** | **Raw Change Score** | **Category** | **Raw Change Score** | **Category** | **Raw Change Score** |
| A* | <0 | A | <0 | A | <0 | A | <0 | A | <0 | A | <0 |
| B | 0-13.76 | B | 0-11.23 | B | 0 | B | 0 | B | 0-19.17 | B | 0-16.75 |
| C | 13.76-20.05 | C | 11.23-16.63 | C | 0-3.93 | C | 0-4.46 | C | 19.17-31.29 | C | 16.75-35.28 |
| D | 20.05-62.59 | D | 16.63-52.59 | D | 3.93-41.8 | D | 4.46-50.33 | D | 31.29-76.3 | D | 35.28-89.98 |
| **Lower Extremity Joint Replacement** | | | | | | | | | | | |
| **IRF-PAI** | | | | **MDS** | | | | **OASIS** | | | |
| **Self-care** | | **Mobility** | | **Self-care** | | **Mobility** | | **Self-care** | | **Mobility** | |
| **Category** | **Raw Change Score** | **Category** | **Raw Change Score** | **Category** | **Raw Change Score** | **Category** | **Raw Change Score** | **Category** | **Raw Change Score** | **Category** | **Raw Change Score** |
| A | <0 | A | <0 | A | <0 | A | <0 | A | <0 | A | <0 |
| B | 0-16.93 | B | 0-15.06 | B | 0 | B | 0 | B | 0-30.85 | B | 0-25.86 |
| C | 16.93-21.69 | C | 15.06-20.73 | C | 0-3.71 | C | 0-4.3 | C | 30.85-38.62 | C | 25.86-35.49 |
| D | 21.69-74.38 | D | 20.73-58.31 | D | 3.71-54.51 | D | 4.3-50.33 | D | 38.62-76.30 | D | 35.49-89.98 |
| **Hip and Femur Fracture** | | | | | | | | | | | |
| **IRF-PAI** | | | | **MDS** | | | | **OASIS** | | | |
| **Self-care** | | **Mobility** | | **Self-care** | | **Mobility** | | **Self-care** | | **Mobility** | |
| **Category** | **Raw Change Score** | **Category** | **Raw Change Score** | **Category** | **Raw Change Score** | **Category** | **Raw Change Score** | **Category** | **Raw Change Score** | **Category** | **Raw Change Score** |
| A | <0 | A | <0 | A | <0 | A | <0 | A | <0 | A | <0 |
| B | 0 | B | 0 | B | 0 | B | 0 | B | 0 | B | 0 |
| C | 0-17.65 | C | 0-14 | C | 0-2.9 | C | 0-4.55 | C | 0-27.53 | C | 0-21.73 |
| D | 17.65-22.24 | D | 14-18.53 | D | 2.9-6.89 | D | 4.55-10.12 | D | 27.53-37.08 | D | 21.73-30.30 |
| E | 22.24-56.20 | E | 18.53-49.66 | E | 6.89-56.20 | E | 10.12-50.33 | E | 37.08-70.65 | E | 30.30-64.12 |

*: A represents the lowest functional group. D represents the highest functional group. IRF-PAI=Inpatient Rehabilitation Facility Patient Assessment Instrument; MDS=Minimum Data Set; OASIS=Outcome and Assessment Information Set.

^: Due to change score calculation, we can only present co-calibrate scores in this table.
