# Supplementary material for: A comparison of three methods in categorizing functional status to predict hospital readmission across post-acute care
Source: PLoS One. 2020 May 7;15(5):e0232017. doi: 10.1371/journal.pone.0232017 (PMC7205206; doi:10.1371/journal.pone.0232017)
Supplement: S5 Table — (DOCX) [file pone.0232017.s005.docx]

**Appendix Table 5. Method III (Functional Staging based on Rasch Model): Raw Scores for IRF-PAI, MDS and OASIS (Self-Care & Mobility) in Stroke, Lower Extremity Joint Replacement and Hip/Femur Fracture.**

| **Stroke** | | | | | | | | | | | |
| --- | --- | --- | --- | --- | --- | --- | --- | --- | --- | --- | --- |
| **IRF-PAI** | | | | **MDS** | | | | **OASIS** | | | |
| **Self-care** | | **Mobility** | | **Self-care** | | **Mobility** | | **Self-care** | | **Mobility** | |
| **Strata** | **Raw Score** | **Strata** | **Raw Score** | **Strata** | **Raw Score** | **Strata** | **Raw Score** | **Strata** | **Raw Score** | **Strata** | **Raw Score** |
| A* | 6-11 | A | 5-15 | A | 5-9 | A | 6-12 | A | 0-1 | A | 0-5 |
| B | 12-26 | B | 16-26 | B | 10-16 | B | 13-28 | B | 2-7 | B | 6-9 |
| C | 27-35 | C | 27-35 | C | 17-26 | C | 29-36 | C | 8-15 | C | 10-14 |
| D | 36-42 |  |  | D | 27-30 |  |  | D | 16-19 |  |  |
| **Lower Extremity Joint Replacement** | | | | | | | | | | | |
| **IRF-PAI** | | | | **MDS** | | | | **OASIS** | | | |
| **Self-care** | | **Mobility** | | **Self-care** | | **Mobility** | | **Self-care** | | **Mobility** | |
| **Strata** | **Raw Score** | **Strata** | **Raw Score** | **Strata** | **Raw Score** | **Strata** | **Raw Score** | **Strata** | **Raw Score** | **Strata** | **Raw Score** |
| A | 6-16 | A | 5-15 | A | 5-10 | A | 6-12 | A | 0-6 | A | 0-9 |
| B | 17-32 | B | 16-35 | B | 6-24 | B | 13-28 | B | 7-12 | B | 10-14 |
| C | 33-42 |  |  | C | 25-30 | C | 29-36 | C | 13-19 |  |  |
| **Hip and Femur Fracture** | | | | | | | | | | | |
| **IRF-PAI** | | | | **MDS** | | | | **OASIS** | | | |
| **Self-care** | | **Mobility** | | **Self-care** | | **Mobility** | | **Self-care** | | **Mobility** | |
| **Strata** | **Raw Score** | **Strata** | **Raw Score** | **Strata** | **Raw Score** | **Strata** | **Raw Score** | **Strata** | **Raw Score** | **Strata** | **Raw Score** |
| A | 6-13 | A | 5-11 | A | 5-9 | A | 6-10 | A | 0-4 | A | 0-3 |
| B | 14-24 | B | 12-25 | B | 10-30 | B | 11-14 | B | 5-9 | B | 4-7 |
| C | 25-42 |  |  |  |  | C | 15-36 | C | 10-15 | C | 8-15 |
|  |  |  |  |  |  |  |  | D | 16-20 |  |  |

*: A represents the lowest functional group. D represents the highest functional group. IRF-PAI=Inpatient Rehabilitation Facility Patient Assessment Instrument; MDS=Minimum Data Set; OASIS=Outcome and Assessment Information Set.
