# Supplementary material for: A comparison of three methods in categorizing functional status to predict hospital readmission across post-acute care
Source: PLoS One. 2020 May 7;15(5):e0232017. doi: 10.1371/journal.pone.0232017 (PMC7205206; doi:10.1371/journal.pone.0232017)
Supplement: S6 Table — (DOCX) [file pone.0232017.s006.docx]

**Appendix Table 6. Demographics and Person-level Characteristics of Inclusion and Exclusion Samples**

|  | **Step 12, before excluding 23% of the potential patients (N=1,001,428)** | |  | | **Step 15, All (N=740,530)** | |
| --- | --- | --- | --- | --- | --- | --- |
|  |  |  |  |  |  |  |
| ***Discharge Locations*** | **IRF** | **SNF** | **HHA** | **IRF** | **SNF** | **HHA** |
| ***Sample Size*** |  |  |  | 137,527 | 325,708 | 277,295 |
| Age, Mean (SD) | 79.3 (7.6) | 80.1 (7.8) | 74.5 (6.4) | 79.0(7.5) | 79.5(7.7) | 74.3(6.2) |
| **Gender** |  |  |  |  |  |  |
| Male | 63,134 (35.3) | 134,051 (27.1) | 132,515 (40.4) | 49,626 (36.1) | 89,454 (27.5) | 114,307 (41.2) |
| Female | 115,813 (64.7) | 360,629 (72.9) | 195,286 (59.6) | 87,901 (63.9) | 236,254 (72.5) | 162,988 (58.8) |
| **Race** |  |  |  |  |  |  |
| Non-Hispanic  White | 151,715 (84.8) | 439,954 (88.9) | 290,297 (88.6) | 116,390 (84.6) | 288,288 (88.5) | 247,947 (89.4) |
| Black | 13,219 (7.4) | 26,707 (5.4) | 17,020 (5.2) | 10,241 (7.5) | 18,288 (5.6) | 13,679 (4.9) |
| Hispanic | 8,641 (4.8) | 15,573 (3.2) | 11,841 (3.6) | 6,747 (4.9) | 10,724 (3.3) | 8,909 (3.2) |
| Others | 5,372 (3.0) | 12,446 (2.5) | 8,643 (2.6) | 4,149(3.0) | 8,408(2.6) | 6,760(2.4) |
| **Total IRF Stay within 90 Days** | 12.5 (5.9) | 0.1 (1.0) | 0.0 (0.3) | 11.8(5.6) | 0.0(0.3) | 0.0(0.2) |
| **Total SNF Stay within 90 Days** | **6.7 (17.4)*** | 24.5 (21.5) | 0.0 (1.2) | 0.3(3.4) | 22.1(17.5) | 0.0(0.8) |
| **Total HH Stay within 90 Days** | 18.6 (23.1) | 13.6 (18.6) | 24.0 (16.2) | 19.8(23.9) | 14.4(18.7) | 22.9(13.4) |
| **Days without staying in IRF, SNF, HHA or long-term care** | 33.2 (31.6) | 33.3 (30.8) | 57.9 (24.4) | 37.5(31.8) | 36.1(30.6) | 60.3(21.8) |
| **Stay in a Hospital/SNF (days)** | 4.3 (2.7) | 4.5 (2.8) | 2.9 (1.6) | 4.2(2.5) | 4.4(2.6) | 2.9(1.5) |
| **Intensive Care (days)** | 1.0 (2.2) | 0.5 (1.8) | 0.2 (0.9) | 0.9(2.1) | 0.5(1.7) | 0.2(0.9) |
| **Coronary Care (days)** | 0.3 (1.2) | 0.2 (1.1) | 0.1 (0.6) | 0.3(1.2) | 0.2(1.0) | 0.1(0.5) |
| **Hierarchical Condition Category Score** | 1.2 (0.7) | 1.1 (0.7) | 0.7 (0.5) | 1.2(0.7) | 1.0(0.7) | 0.7(0.5) |
| **Comorbidity (based on Elixhauser Comorbidity Index)** | 3.4 (1.9) | 3.0 (1.9) | 2.2 (1.6) | 3.4(1.9) | 3.0(1.9) | 2.2(1.6) |
| 0 | 5,613 (3.14) | 25,469 (5.2) | 35,466 (10.8) | 4,513 (3.3) | 17,826 (5.5) | 30,294 (10.9) |
| 1-3 | 96,118 (53.7) | 294,096 (59.5) | 229,952 (70.2) | 75,148 (54.6) | 197,692(60.7) | 196,382(70.8) |
| 4-6 | 65,693 (36.7) | 150,160 (30.4) | 57,021 (17.4) | 49,333(35.9) | 95,028(29.2) | 46,575(16.8) |
| ≥7 | 11,523 (6.4) | 24,928 (5.0) | 5,373 (1.6) | 8,533(6.2) | 15,162(4.7) | 4,044(1.5) |
| **Diagnosis** |  |  |  |  |  |  |
| *Stroke* |  |  |  |  |  |  |
| Ischemic | 68,076 (38.0) | 62,584 (12.7) | 40,929 (12.5) | 50,549 (36.8) | 36,168 (11.1) | 30,168 (10.9) |
| Hemorrhagic | 8,554 (4.8) | 8,510 (1.7) | 3,880 (1.2) | 6,004 (4.4) | 4,864 (1.5) | 2,917 (1.1) |
| *Lower Extremity Joint Replacement* |  |  |  |  |  |  |
| Elective | 13,210 (7.4) | 70,643 (14.3) | 84,863 (25.9) | 11,273 (8.2) | 52,301 (16.1) | 73,190 (26.4) |
| Non-Elective | 24,562 (13.7) | 70,982 (14.4) | 8,118 (2.5) | 19,333 (14.1) | 44,036 (13.5) | 6,443 (2.3) |
| Knee | 25,113 (14.0) | 153,722 (31.1) | 176,260 (53.8) | 21,779 (15.8) | 113,345(34.8) | 154,205 (55.6) |
| Others | 544 (0.3) | 1,944 (0.4) | 1,114 (0.3) | 438(0.3) | 1,291(0.4) | 942 (0.3) |
| *Hip and Femur Fracture* |  |  |  |  |  |  |
| Femur | 3,619 (2.0) | 13,856 (2.8) | 1,385 (0.4) | 2,522(1.8) | 7320(2.3) | 948 (0.3) |
| Femur Neck | 34,238 (19.1) | 108,443 (21.9) | 10,211 (3.11) | 24,886 (18.1) | 64,112 (19.7) | 7,672 (2.8) |
| Complications | 643 (0.4) | 2,752 (0.6) | 638 (0.2) | 463(0.3) | 1,543(0.5) | 492 (0.2) |
| Others | 544 (0.3) | 1,944 (0.4) | 1,114 (0.3) | 280(0.2) | 728(0.2) | 318 (0.1) |
| **Disability at Original Entitlement** |  |  |  |  |  |  |
| Yes | 25,312 (14.1) | 76,040 (15.4) | 24,399 (7.4) | 13,608 (9.9) | 28,709 (8.8) | 22,231 (8.0) |
| No | 161,907 (90.5) | 450,844 (91.1) | 303,402 (92.6) | 123,919 (90.1) | 296,999 (91.2) | 255,064 (92.0) |
| **Medicaid Eligibility** |  |  |  |  |  |  |
| Yes | 25,312 (14.1) | 76,040 (15.4) | 24,399 (7.4) | 17,952 (13.1) | 43,417 (13.3) | 18,385 (6.6) |
| No | 153,635 (85.9) | 418,640 (84.6) | 303,402 (92.6) | 119,575 (86.9) | 282,291 (86.7) | 258,910 (93.4) |
| **30-Day Readmission** | 17,465 (9.8) | 44,277 (9.0) | 15,005 (4.6) | 14,995 (10.9) | 30,815 (9.5) | 9,803 (3.5) |
| **90-Day Readmission** | 34,874 (19.5) | 85,228 (17.2) | 30,892 (9.4) | 26,210 (19.1) | 58,407 (17.9) | 22,298 (8.0) |

*: Significant at p<0.05 level. IRF=inpatient rehabilitation facility; SNF=skilled nursing facility; HHA=home health agency.
