# Supplementary material for: A comparison of three methods in categorizing functional status to predict hospital readmission across post-acute care
Source: PLoS One. 2020 May 7;15(5):e0232017. doi: 10.1371/journal.pone.0232017 (PMC7205206; doi:10.1371/journal.pone.0232017)
Supplement: S1 Fig — (DOCX) [file pone.0232017.s007.docx]

**Appendix Figure 1. Method I: Use Percentile Admission Score to Generate Functional Score Categories (Example of IRF-PAI Self-Care in Stroke).**

Six self-care items: Eating, grooming, bathing, dressing-upper, dressing-lower, toileting

**Step 1**

Sum-up Self-Care score (six items) at admission of IRF-PAI for Stroke

**Tertile**: 0-33%, 33-66%, 66-100%; **Quartile**: 0-25%; 25%-50%, 50-75%, 75-100%; **Quintile**: 0-20%, 20-40%, 40-60%, 60-100%

Run tertile, quartile and quintile separately for IRF, SNF and HHA.

Based on the distribution of total Self-Care summed raw score, run separately for tertile, quartile and quintile to determine cutoff scores

Use C-statistics to determine the relatively optimal proportional category for this domain (see box on the right).

C-statistics Comparisons:

Tertile: 0.6238 (c-statistics)

Quartile: 0.6274 (c-statistics)

Quintile: 0.6266 (c-statistics)

Generating four categories of Self-Care score in IRF-PAI for Stroke

Four categories based on quartile of admission scores of Self-Care in IRF-PAI for stroke (from **lowest (A)** to **highest (D)** function): *converted raw score from 0-100 scale

**Category A**: 6~14 (raw score*, as 0-25%) or 16.80~42.04 (co-calibrated 0-100 scale)

**Category B**: 15~20 (raw score, as 25-50%) or 43.46~49.63 (co-calibrated 0-100 scale)

**Category C**: 21~25 (raw score, as 50-75%) or 50.74~55.03 (co-calibrated 0-100 scale)

**Category D**: 26~42 (raw score, as 75-100%) or 56.11~91.18 (co-calibrated 0-100 scale)

Determine using **quartile** of Self-Care in IRF-PAI for Stroke

**Step 4**

**Step 2**

**Step 3**
