# Supplementary material for: A comparison of three methods in categorizing functional status to predict hospital readmission across post-acute care
Source: PLoS One. 2020 May 7;15(5):e0232017. doi: 10.1371/journal.pone.0232017 (PMC7205206; doi:10.1371/journal.pone.0232017)
Supplement: S2 Fig — (DOCX) [file pone.0232017.s008.docx]

**Appendix Figure 2. Method II: Use Percentile Change Score to Generate Functional Score Categories (Example of IRF-PAI Self-Care in Stroke).**

Six self-care items: Eating, grooming, bathing, dressing-upper, dressing-lower, toileting

Calculate change score of six Self-Care items between admission and discharge of IRF-PAI (Stroke)

**Step 1**

**Tertile**: 0-33%, 33-66%, 66-100%; **Quartile**: 0-25%; 25%-50%, 50-75%, 75-100%; **Quintile**: 0-20%, 20-40%, 40-60%, 60-100%

Run tertile, quartile and quintile separately for IRF, SNF and HHA.

Determine tertile, quartile and quintile based on the change score distributions.

**Step 2**

Use C-statistics to determine the relatively optimal proportional category to use in the final model (Model 4)

C-statistics Comparisons: Tertile: 0.6114; Quartile: 0.6118; Quartile: 0.6117

Four categories based on change score of Self-Care in IRF-PAI for stroke (from **lowest (A)** to **highest (D)** function): *below are co-calibrated score based on 0-100 scale. As change score was not able to converted back to the raw score.

**Category A**: <0

**Category B**: >=0 & <13.76

**Category C**: >=13.76 & <20.05

**Category D**: >=20.05 & <62.59

Operational Definition:

***Tertile***: Negative to small positive change, medium positive and large positive change.

***Quartile***: Negative and zero change, small, medium and large positive change.

***Quintile***: Negative change, zero change, small, medium and large positive change

Replace change score cutoff values with operational defined cutoff values.

Determine using **quartile** of Self-Care in IRF-PAI for Stroke

**Step 5**

**Step 4**

**Step 3**

Example:

***Tertile***: <=10.35, >10.35 & <=17.78, >17.78 & <=62.59.

***Quartile***: <0, >0 & <=13.76, >13.76 & <=20.05, >20.05 & <= 62.59.

***Quintile***: <0, =0, >0 & <=15.96, >15.96 & <=21.55, >21.55 & <=62.59.

Note: Using a combination of tertile, quartile and quintile and operational definitions to decide the cutoff.
